# The Relative Contribution of Proximal 5′ Flanking Sequence and Microsatellite Variation on Brain Vasopressin 1a Receptor (*Avpr1a*) Gene Expression and Behavior

**DOI:** 10.1371/journal.pgen.1003729

**Published:** 2013-08-29

**Authors:** Zoe R. Donaldson, Larry J. Young

**Affiliations:** 1 Division of Integrative Neuroscience, Department of Psychiatry, Columbia University, New York, New York, United States of America; 2 Center for Translational Social Neuroscience, Department of Psychiatry and Behavioral Sciences, Yerkes National Primate Research Center, Emory University, Atlanta, Georgia, United States of America; University of Arizona, United States of America

## Abstract

Certain genes exhibit notable diversity in their expression patterns both within and between species. One such gene is the vasopressin receptor 1a gene (*Avpr1a*), which exhibits striking differences in neural expression patterns that are responsible for mediating differences in vasopressin-mediated social behaviors. The genomic mechanisms that contribute to these remarkable differences in expression are not well understood. Previous work has suggested that both the proximal 5′ flanking region and a polymorphic microsatellite element within that region of the vole *Avpr1a* gene are associated with variation in V1a receptor (V1aR) distribution and behavior, but neither has been causally linked. Using homologous recombination in mice, we reveal the modest contribution of proximal 5′ flanking sequences to species differences in V1aR distribution, and confirm that variation in V1aR distribution impacts stress-coping in the forced swim test. We also demonstrate that the vole *Avpr1a* microsatellite structure contributes to *Avpr1a* expression in the amygdala, thalamus, and hippocampus, mirroring a subset of the inter- and intra-species differences observed in central V1aR patterns in voles. This is the first direct evidence that polymorphic microsatellite elements near behaviorally relevant genes can contribute to diversity in brain gene expression profiles, providing a mechanism for generating behavioral diversity both at the individual and species level. However, our results suggest that many features of species-specific expression patterns are mediated by elements outside of the immediate 5′ flanking region of the gene.

## Author Summary

DNA sequence variation underlies many differences both within and between species. In this paper, we investigate a specific DNA sequence that is thought to influence expression of a gene that modulates behavior, the vasopressin V1a receptor gene (*Avpr1a*). Specifically, differences in the expression of V1a receptor in the brain have been causally tied to social behavior differences, but the genetic basis of these differences is not understood. Using transgenic mice, we investigate the role of DNA sequences upstream of this gene in generating species-specific and individual variation in *Avpr1a* expression. We find that, contrary to our expectation, this region has only a modest influence on differences in expression patterns across rodent species. This indicates that DNA elements outside of this region play a larger role in species-level differences in expression. We confirm that variation in *Avpr1a* expression mediated by this upstream region translates to differences in behavior. We also find that variable DNA sequences associated with repetitive motifs within this region subtly influence gene expression. Together these findings highlight the complexity of genetic mechanisms that influence diversity in brain receptor patterns and support the idea that variable repetitive elements can influence both species and individual differences in gene expression patterns.

## Introduction

The genomic mechanisms that give rise to phenotypic diversity across species or among individuals within a species are not well understood. Behavior is a trait that is particularly well suited for exploring genetic mechanisms underlying phenotypic plasticity, as it is an evolutionarily labile trait. Social behaviors, in particular, can be markedly variable among closely related species, and often display significant individual variability within a species [1]–[6]. Genomic mechanisms that give rise to diversity in behavior fall into two categories; those that alter protein structure and function (e.g. coding region mutations) and those that alter the expression of genes [7], [8]. In *Caenorhabditis elegans*, for example, variation in a single nucleotide of *npr-1*, which alters the neuropeptide receptor *protein structure*, has been shown to be responsible for strain differences in social feeding behavior [9]. However, it is likely that a significant portion of phenotypic diversity is derived from mutations that alter gene expression [10]–[13]. Sequences in the 5′ flanking region of genes regulate tissue-specific expression in many cases, and are thus likely candidates for contributing to species-specific expression patterns. In addition, unstable, polymorphic repetitive elements surrounding genes have been proposed as a mechanism to enhance evolvability of traits by increasing diversity in gene expression [14], [15]. The vasopressin 1a receptor gene (*Avpr1a*) provides an excellent opportunity to explore both of these potential mechanisms of gene expression divergence [16], [17].

Arginine vasopressin (AVP) is an evolutionarily conserved neuropeptide that modulates a wide range of behaviors including stress coping, territorial aggression, mate-guarding, pair bonding and paternal care [18]–[21]. The vasopressin 1a receptor (V1aR) is a G-protein coupled receptor that mediates many of the behavioral effects of AVP [22]. While the structure and brain distribution of AVP are highly conserved among mammals, the behavioral effects of this peptide, and the neural distribution of V1aR vary markedly across species [23]–[25]. Among voles, for example, AVP facilitates affiliative behavior and selective aggression related to pair bonding in monogamous prairie voles (*Microtus ochrogaster*), but not in the closely related, non-monogamous montane voles (*M. montanus*) [24], [26]. Accompanying these species differences in behavioral response to AVP are remarkable species differences in V1aR distributions in the brain. For example, monogamous prairie voles have higher densities of V1aR in the ventral pallidum, central amygdala, and dentate gyrus than nonmonogamous montane or meadow voles (*M. pennsylvanicus*) [1], [27], [28]. These differences in V1aR distribution are due to species differences in the expression of the *Avpr1a* gene [26]. Furthermore, pharmacologically blocking ventral pallidal V1aR prevents mating-induced partner preference formation in prairie voles [29]. These observations suggest that variation in the neural expression patterns of *Avpr1a* may underlie species differences in AVP-dependent behaviors.

There is substantial direct evidence supporting the hypothesis that diversity in expression of *Avpr1a* within the brain contributes to both intra-and inter-specific differences in behavior. For example, increasing V1aR density in the lateral septum using viral vector mediated gene transfer enhances social recognition memory in rats while increasing V1aR density in the ventral pallidum of prairie voles facilitates affiliation and pair-bond formation [30], [31]. In addition, increasing V1aR density in the anterior hypothalamus increases selective aggression in prairie voles [32]. Relatively subtle variation in expression can profoundly affect behavior since viral vector mediated RNA interference in the ventral pallidum, which results in a 30% reduction in V1aR binding, significantly reduces pair bonding behavior in prairie voles [33]. Remarkably, increasing V1aR in the ventral pallidum of meadow voles using a viral vector to mimic the distribution of V1aR in the prairie vole brain confers the ability to form a partner preference in this promiscuous species [34]. Likewise, transgenic mice carrying the prairie vole *Avpr1a* locus display a pattern of V1aR binding similar to that of prairie voles, and this difference in receptor patterns leads to increased affiliative behavior in response to AVP [24]. These experiments demonstrate conclusively that diversity in *Avpr1a* expression within the brain directly contributes to both inter and intra-species variability in AVP-mediated behaviors. Here we explore the contribution of both the 5′ flanking region and variation in hypermutable microsatellite sequences within this region in the generation of this variability in gene expression.

**Figure 1:**
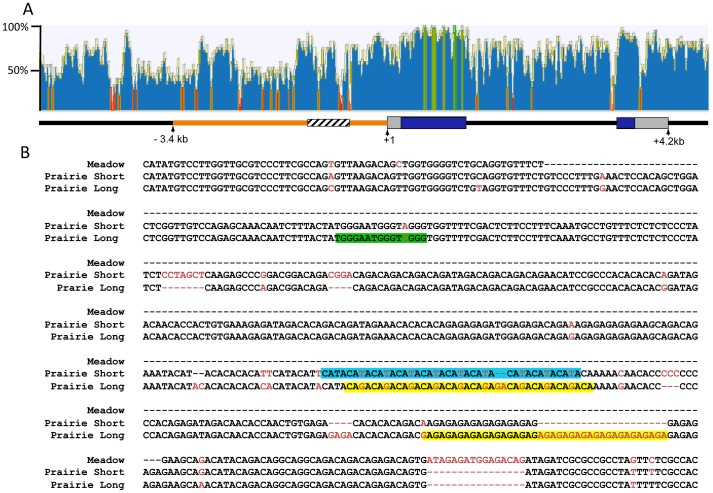
Comparison of the mouse and vole *Avpr1a* locus. ClustalW was used to align 10.8 kb of mouse and prairie vole sequence containing the *Avpr1a* gene and sequence identity was calculated using a sliding 30 bp window using Geneious software (A). Green indicates areas of 100% identical sequence while red areas have <30% sequence identity between vole and mouse. The microsatellite region is shown in cross hatch and the 5′ flanking region targeted for replacement is shown in orange. The pairwise percent identity of the replaced 5′ flanking region between prairie vole and mouse is 53.6%. (B) Voles have a complex microsatellite element upstream of *Avpr1a* (crosshatched region; A) that exhibits both species and individual differences in sequence composition and length. The alignment of the meadow and prairie microsatellite alleles used in our targeting vectors is shown. Sequence differences are shown in red, and potential differential transcription factor bindings sites have been shaded. Green = Rreb1 binding site unique to the long allele; blue and yellow regions indicate differential binding opportunities for factors recognizing TATA-like and GAGA-like sequences, respectively. Although not shown, the montane vole has the same general structure as the meadow vole with regard to the microsatellite.

The 2.2 kb of sequence upstream of the *Avpr1a* transcription start site have been hypothesized to contain regulatory sequences that contribute to the prairie vole-like patterns of V1aR [24]. Transgenic mice carrying a randomly inserted prairie vole *Avpr1a* transgene, comprised of 2.2 kb of 5′ flanking sequence, exons, introns and some downstream sequences from the prairie vole, displayed a receptor pattern more similar to that of a prairie vole than a mouse [24]. However, this prairie vole-like pattern was found in only one of four independently derived transgenic mouse lines carrying identical transgenes, suggesting that the integration site within the genome had a strong impact on expression pattern and raising a question as to the extent to which this region is actually responsible for species-specific expression patterns. Within this 5′ flanking region is a series of variable nucleotide tandem repeats (VNTRs) interspersed with non-repetitive DNA, known as the *Avpr1a* microsatellite. This microsatellite lies ∼760 bp upstream of the *Avpr1a* transcription start site and displays polymorphisms in repeat numbers and sequence content across and within vole species, and thus represents a “hot spot” for mutations (Figure 1) [17], [35].

Both species and individual differences in the *Avpr1a* microsatellite are sufficient to drive differences in gene expression in a cell-type specific manner *in vitro*, suggesting that this genetic region may represent a source of both inter- and intraspecies receptor expression variation [17], [35]. This hypothesis is further supported by associations between microsatellite length, receptor patterns, and social behavior in prairie voles [17], [36]. Specifically, in a laboratory setting, male prairie voles selectively bred to have long *Avpr1a* microsatellites were more likely to form partner preferences than males with short microsatellites [17]. Furthermore, long and short *Avpr1a* microsatellite prairie voles have different patterns of V1aR distribution in the brain [35]–[37]. However, this selection experiment cannot distinguish between the contribution of the microsatellite and other linked functional genetic variants that affect expression. Remarkably, similar polymorphisms in *AVPR1A* microsatellites have been associated with gene expression, brain activation, and social behavior in humans and chimpanzees [38]–[41]. Thus the vole *Avpr1a* is an ideal model locus for exploring the genomic mechanisms contributing to diversity in brain gene expression and behavior that has relevance to human behavior. More specifically, *Avpr1a* provides an opportunity to explore the relative contribution of species-specific regulatory elements in the 5′ flanking region and of polymorphic repetitive elements in that region in generating species-specific patterns and individual variation in brain gene expression.

We hypothesized that replacing 3.4 kb of the 5′ flanking region of the mouse *Avpr1a* gene with the prairie vole homologue would result in a prairie vole-like pattern of V1aR binding. We further hypothesized that variation in the microsatellite element in this region would confer variation in receptor distribution. To test these hypotheses, we used homologous recombination to create three lines of knock-in mice in which 3.4 kb of the mouse 5′ flanking region was replaced with the corresponding prairie vole sequence. Each line differed only with regard to the microsatellite element – either from meadow vole or the prairie vole long or prairie vole short variants that had previously been associated with individual variation in V1aR distribution. Using receptor autoradiography, we assessed the contribution of the 5′ flanking region for determining species-specific V1aR expression patterns and microsatellite variability in generating variation in V1aR expression patterns *in vivo* in these three lines of mice. Our results demonstrate that both inter- and intra-species variability in the microsatellite confers differences in receptor binding in the thalamus, amygdala, and dentate gyrus, mirroring naturally-occurring differences observed both between and within vole species. However, the 5′ flanking region is not sufficient to confer species-typical binding patterns, but is sufficient to quantitatively change expression levels in a direction consistent with species differences in binding. Based on these results, we determined whether the observed differences in expression led to behavioral differences, and found that alterations in receptor binding are associated with differences in coping strategy in the forced swim test but not with differences in learning and memory in the novel object recognition task.

## Results

### Generation of *Avpr1a* knock-in mice

We used recombinant transgenic technology to replace the 5′ flanking region of the mouse *Avpr1a* gene with corresponding sequence from the prairie vole. We chose to replace 3.4 kb because this was larger than the 2.2 kb previously used to generate a traditional transgenic mouse through pronuclear injection [24] and contained a high density of low sequence homology (53.6% identity between mouse and prairie vole) while still being small enough for efficient homologous recombination (Figure 1a). We hypothesized that this sequence divergence would contain the elements that confer species-specific expression patterns. In order to also investigate the hypothesis that microsatellite variation within this region may contribute to species and individual differences in expression patterns, we generated 3 lines that differed only in the content of one of three *Avpr1a* microsatellites – either the meadow version, or a long or short version from the prairie vole (Figure 1b) - within the same prairie vole 5′ flanking region. The meadow microsatellite cassette was 175 bp long, and the short and long prairie alleles were 608 and 623 bp long, respectively.

**Figure 2:**
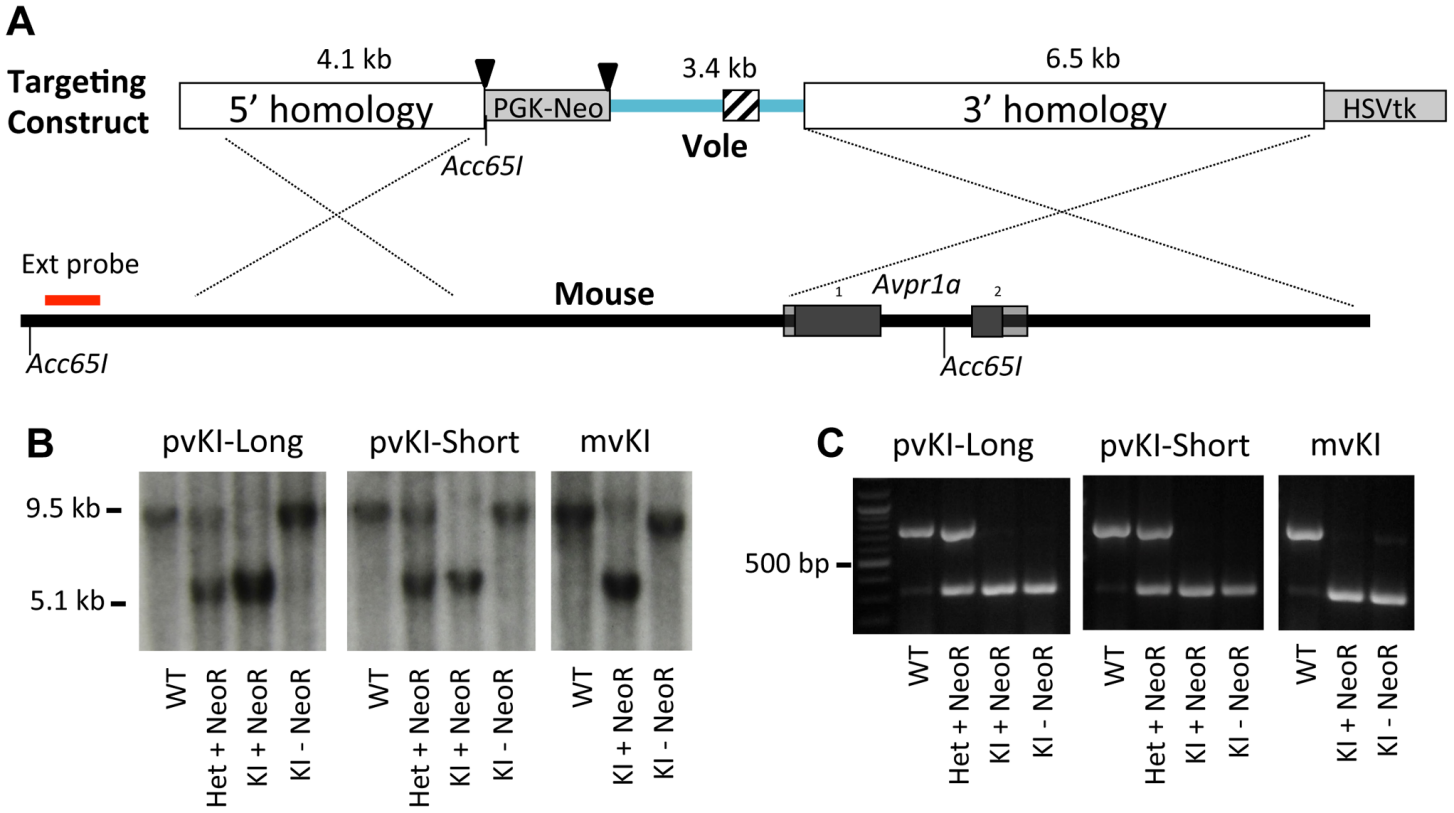
Targeting vector design. (A) shows the targeting vector used to replace the 5′ flanking region of the mouse *Avpr1a* gene with corresponding sequences from the prairie vole. We generated three targeting vectors that were identical except for the microsatellite region they contained, which is indicated by the cross hatched region. Triangles denote loxP sites. (B) shows hybridization of the external Southern probe in correctly targeted recombinants for all three lines. Because the *Acc651* site used to screen for recombinant stem cells was located within the floxed region, excision of the NeoR also resulted in the recombinant allele yielding a ∼9.5 kb band when detected with the external probe. (C) shows PCR genotyping. All three lines were backcrossed to a C57Bl/6J background for at least 5 generations prior to neuroanatomical and behavioral experiments.

The targeting strategy is illustrated in Figure 2. For the meadow line, we screened 288 ES cell clones and identified 1 recombinant. For the prairie short line, 192 clones yielded 2 correct recombinants, and for the prairie long line, 288 clones yielded 2 correct recombinants. This corresponds with an overall recombination efficiency of 0.6% (5 of 768). The floxed PGK-NeoR cassette was successfully removed via breeding to a ubiquitously expressing EIIa-Cre recombinase line as confirmed by PCR and Southern Blot (Figure 2). Because the *Acc651* site used to screen for recombinant stem cells was located within the floxed region, excision of the NeoR also resulted in the recombinant allele yielding a ∼9.5 kb band when detected with the external probe. The three resulting recombinant alleles, prairie vole long (pvKI-long), prairie vole short (pvKI-short), or meadow vole (mvKI) were identical in sequence except for the composition of the microsatellite element. All three lines were backcrossed to a C57Bl/6J background for at least 5 generations prior to neuroanatomical and behavioral experiments.

Because we performed recombination in hybrid B6/129 ES cells, there was a possibility that recombination could occur at either the C57Bl6/J or the 129SvEv locus. In order to determine the integration site for our three lines, we genotyped rs13480799, which is located outside of the 5′ homology arm upstream of the *Avpr1a* gene. This SNP is a G in most lines examined, including C57-related lines, but is a C in 129-related lines [42]. Sequencing revealed that the targeting construct recombined in the C57Bl6/J allele in the mvKI line, and into the 129SvEv alleles in both pvKI lines. While this represents a potential confound that should be taken into account when considering our results, C57Bl6/J and 129SvEv strains differ very little at this locus. When comparing C57-related (C57Bl6/J and C57L/J) and 129-related (129S1/SvImJ and 129X1/SvJ) strains within 100 kb surrounding the Avpr1a locus (Chr10:121850000–121950000; NCBI37/mm9), only 5 known SNP differences (rs29315655, rs29348001, rs13480799, rs29342115, rs633704) and 1 unresolved potential difference (rs29379744) have been described in the JAX Mouse Genome Informatics SNP database [42], [43]. 28 SNPs have been described across all strains for this region. Thus while it is possible that our line differences could be attributable to the strain origin of the locus of recombination, it is unlikely because these mouse strains are so similar in this region.

### Contribution of the *Avpr1a* proximal 5′ flanking regions to species-specific V1aR patterns

Previous work had suggested that some of the elements integral to species-specific neural V1aR patterns existed within the 2.2 kb upstream of the transcription start site of the *Avpr1a* gene [24]. However, independently derived lines of transgenic mice carrying this region displayed different expression patterns due to differences in chromosomal integration of the transgene. Furthermore, those transgenes also contained coding regions, introns and 3′ flanking sequences. In order to more precisely explore the role of the 5′flanking region in guiding species-specific V1aR patterns, we compared V1aR binding (as a proxy for *Avpr1a* expression) in wildtype (WT) and pvKI-long littermates at post-natal day (PND) 60–70. Because the endogenous mouse 5′ flanking region was replaced with prairie vole sequence, this technique was not subject to random integration effects, as occurs in traditional pronuclear injection transgenics.

**Figure 3:**
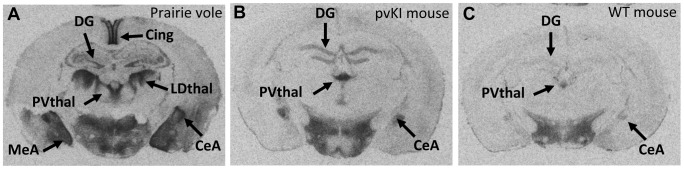
The 5′ flanking region of *Avpr1a* contributes to neural V1aR expression patterns. In order to determine whether the replaced 5′ flanking region influences V1a receptor patterns, we compared brains of pvKI-long mice (B) with those of WT mice (C). pvKI-long mice display V1a patterns that contain elements of both mice and prairie vole patterns (A). Specifically, mice carrying the prairie vole promoter region displayed increased levels of V1a receptor in the dentate gyrus (DG), paraventricular nucleus of the thalamus (PVthal), and the central amygdala (CeA), but not in the ventral pallidum (VP), cingulate cortex (Cing), laterodorsal thalamus (LDthal), or medial amygdala (MeA).

We used V1aR autoradiography as a proxy for *Avpr1a* gene expression since this technique is much more quantitative and sensitive than *in situ* hybridization, provides greater anatomical resolution than qPCR, and accurately reflects *Avpr1a* mRNA patterns (Young 1997). Furthermore, since the replaced region lies upstream of the transcription start site, variation in that sequence should not affect post-transcriptional processing. The greater signal to noise ratio of this technique allows us to detect relatively subtle differences in V1aR protein binding. Replacement of the 5′ flanking region of the murine *Avpr1a* locus with the same region from prairie voles yielded qualitative patterns of V1aR with elements of both mouse and prairie vole expression (Figure 3). To initially explore the effects of our manipulation on V1aR levels, we performed an overall ANOVA with three factors: genotype (WT, mvKI, pvKI-short, and pvKI-long), brain region, and sex. We identified main effects of genotype (F(3, 137) = 35.7, p<0.001), brain region (F(4, 137) = 1035.1, p<0.001), and sex (F(1, 137) = 10748.9, p<0.001). In addition, there were genotype×brain region (F(12, 137) = 8.902, p<0.001) and genotype×sex interactions (F(3, 137) = 3.451, p = 0.02), but no brain region×sex (F(4, 137) = 2.0, p = 0.09) or genotype×brain region×sex (F(12, 137) = 1.425, p = 0.16) interactions. Therefore, we did not analyze sex differences for each brain region in each of the lines. The main effect of sex appeared to be driven by the fact that females tend to have slightly higher levels of V1aR binding in some brain regions. However, since there are equal numbers of males and females across groups, and our focus was on the impact of promoter elements on expression, we collapsed males and females into a single group. We then performed three separate ANOVAs to test the a priori hypotheses regarding 1) the role of the 5′ flanking region, 2) species differences in the microsatellite, and 3) intraspecies differences in the microsatellite. To address the first of these, we compared pvKI-long mice homozygous for this region with WT mice. pvKI-long mice showed an increase in V1aR binding in the ventral pallidum (VP), central amygdala (CeA), paraventricular nucleus of the thalamus (PVthal), and dentate gyrus (DG) of the hippocampus, consistent with the distribution in the prairie vole. Specifically, we compared V1aR binding in pvKI-long mice to WT mice, and identified a significant effect of both brain region (F(4, 100) = 511.6, p<0.001) and genotype (F(4, 100) = 79.2, p<0.001) on V1aR levels. In addition, there was a significant interaction between brain region and genotype (F(4, 100) = 10.6, p<0.001), and simple main effects with Sidak-adjusted **α** showed that pvKI-long animals had significantly higher levels of V1aR than WT mice in the VP (p<0.001), CeA (p<0.001), PVThal (p<0.001), and DG (p<0.001), but not in the lateral septum (LS; p = 0.24; Figure 3). While it is intriguing that there is a significant difference in binding in the VP of these two lines, the actual difference is quite modest (on average, 1.15 fold higher). In addition, it should be noted that the binding in the dentate gyrus was distributed differently between prairie voles compared to pvKI-long mice, potentially due to different effects of the mouse versus vole coding sequences on receptor trafficking. However, the pvKI-long mice did not show the prairie vole specific binding in the laterodorsal thalamus (LDthal) or medial amygdala (MeA) (Figure 3). The overall similarities in binding pattern between the pvKI-long and WT mice demonstrate that elements outside of the replaced element (e.g. distal 5′ flanking regions, introns, and other surrounding elements) contribute significantly to species-typical expression, perhaps more so than the sequences in the 3.4 kb regions that we tested. However, the quantitative differences between these two lines of mice demonstrate conclusively that the proximal 3.4 kb of the 5′ flanking region also contributes to species-specific patterns of V1aR distribution in the brain.

### Role of the *Avpr1a* microsatellite in directly modulating species-specific patterns of V1aR

**Figure 4:**
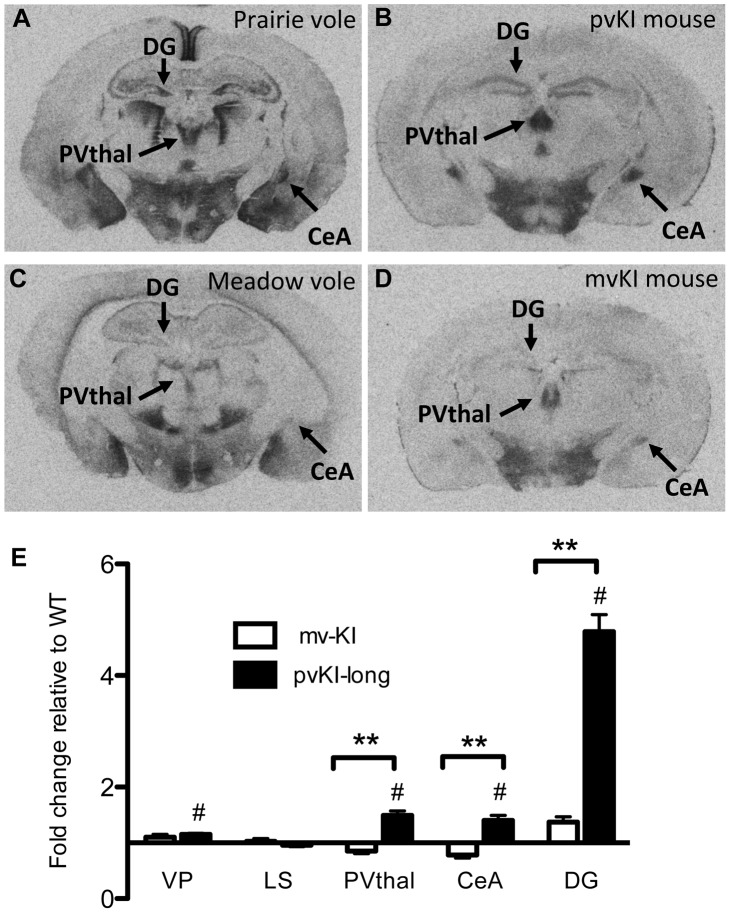
Microsatellite differences modulate species differences in V1aR patterns. Mice carrying the prairie vole *Avpr1a* microsatellite (B), as compared to mice carrying the meadow microsatellite (D), have higher V1aR binding in the dentate gyrus (DG), paraventricular nucleus of the thalamus (PVThal), and the central amygdala (CeA). These differences mirror those observed in the same brain regions of prairie (A) and meadow voles (C). (E) shows the difference in V1a levels relative to WT mice. Data are represented as mean ± SEM; n = 7–8 animals/group; **p<0.001, #p<0.05 compared to WT.

Having established that replacement of the 5′ flanking region contributes to differences in V1aR levels in the thalamus, amygdala, ventral pallidum, and hippocampus, we next investigated whether differences in the composition of the *Avpr1a* microsatellite might mediate species differences in V1aR binding within any of these regions. Specifically we compared V1aR binding in KI mice homozygous for either the prairie long (pvKI-long) or meadow vole (mvKI) *Avpr1a* microsatellite, and identified a significant effect of both brain region (F(4, 77) = 114.7, p<0.001) and genotype (F(4, 77) = 160.9, p<0.001) on V1aR levels. In addition, there was a significant interaction between brain region and genotype (F(4, 77) = 74.2, p<0.001), and simple main effects with Sidak-adjusted **α** showed that pvKI-long animals had significantly higher levels of V1aR than mvKI animals in the CeA (p<0.001), PVThal (p = 0.002), and DG (p<0.001), but not in the lateral septum (LS; p = 0.68; Figure 4E) or ventral pallidum (VP; p = 0.75; Figure 4E).

Although data are not available directly comparing V1aR in these brain regions in meadow and prairie voles, meadow voles have an expression pattern that is similar to that of montane voles, and that comparison has previously been examined [27]. Table 1 shows the ratio of expression calculated for prairie∶montane voles derived from the binding values reported in table 1 from Wang et al. [44] compared with the binding ratio of the same regions in pvKI-long∶mvKI mice. These studies are independent and warrant care in drawing parallels, but overall, these ratios indicate that the binding differences between vole species are broadly mirrored in the thalamus, CeA, and DG but not the VP or LS in these mouse lines.

**Table 1:** Comparison of V1aR binding ratios in prairie∶montane voles and pvKI-long∶mvKI mice.

Brain region	Prairie Vole∶Montane Vole	pvKI-long∶mvKI
Ventral Pallidum+	1.51§	1.05
Lateral Septum	0.37§	0.94
Thalamus*	1.69§	1.76§
Central Amygdala	3.15§	1.79§
Dentate Gyrus	2.32§	3.50§

+ ratio calculated for region incorrectly identified as diagonal band by Wang et al. [27].

* ratio calculated for region identified as mediodorsal thalamus in [27].

§ indicates significant differences between species or between mouse lines (p<0.05).

### Contribution of intra-specific variation in *Avpr1a* microsatellite structure on V1aR distribution

**Figure 5:**
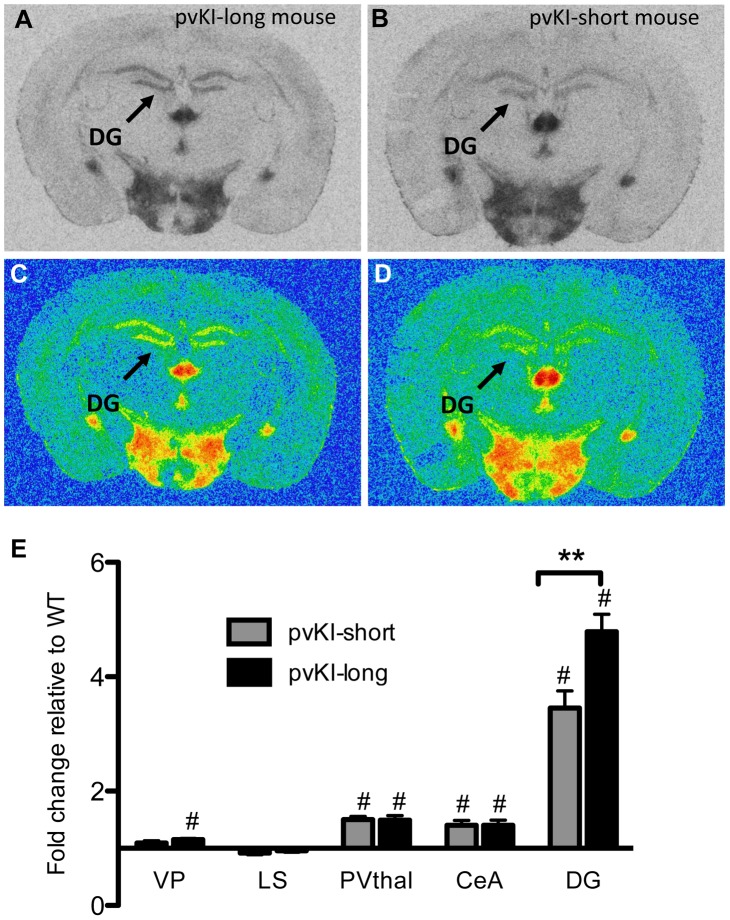
Allelic differences in the *Avpr1a* microsatellite contribute to intraspecies variation in V1aR binding. Comparison of mouse lines homozygous for either the long (A, C) or short version (B, D) of the *Avpr1a* microsatellite showed that mice carrying the long version had higher levels of V1a in the dentate gyrus (DG) (E). Data are represented as mean ± SEM; n = 8 animals/group; **p<0.01, #p<0.05 compared to WT.

Allelic variation in *Avpr1a* has also been tied to intra-species variation in V1aR patterns in prairie voles [17], [36], [37]. However, these studies could not distinguish direct effects of the microsatellite from the possibility that the microsatellite is in linkage disequilibrium with other functional elements. In order to determine the direct contribution of the microsatellite to individual differences in neural V1aR distributions, we compared V1aR binding patterns in mice homozygous for either the long (pvKI-long) or the short version (pvKI-short) of the prairie vole microsatellite in the VP, LS, CeA, PVthal, and DG. The prairie long and short form of the microsatellite are substantially more similar to each other than to the meadow microsatellite. As such, we predicted that the potential differences conferred by this region would be relatively subtle. A 2-way ANOVA revealed a main effect of both brain region (F(4, 80) = 165.0, p<0.001) and genotype (F(4, 80) = 12.1, p = 0.001) on V1aR levels (Figure 5E). In addition, there was a significant interaction between brain region and genotype (F(4, 80) = 7.8, p<0.001), and simple main effects analysis with Sidak-adjusted **α** showed that pvKI-long mice had higher V1aR levels in the DG (p<0.01) but not in the CeA (p = 0.42), PVThal (p = 0.96), LS (p = 0.93) or VP (p = 0.77; Figure 5). Although significant for the DG, the differences observed between these mice are less profound than reported for prairie voles with different microsatellite lengths, suggesting that while individual differences in microsatellite structure do directly impact expression in the brain, other linked polymorphisms may account for the larger number of regional differences found in prairie voles.

### Impact of variation of *Avpr1a* expression on learning and stress-coping behaviors

While there is considerable evidence in mice and voles that variation in *Avpr1a* expression has behavioral consequences [24], [31], [32], [34], [45], we wanted to determine whether the variation in V1aR distribution in our KI lines contributed to variation in behavior. V1aR activation modulates a wide array of behaviors, and we used the existing literature on the role of V1aR and our binding data to guide our behavioral investigation. While variation in V1aR distribution in voles has been studied extensively with respect to social behavior, the brain regions showing line differences in our mice have not been implicated in regulating AVP-dependent social behaviors. Instead, we focused on changes in the hippocampus and the CeA — regions in which AVP and V1aR function has previously been studied in rats and mice [46]–[49].

**Figure 6:**
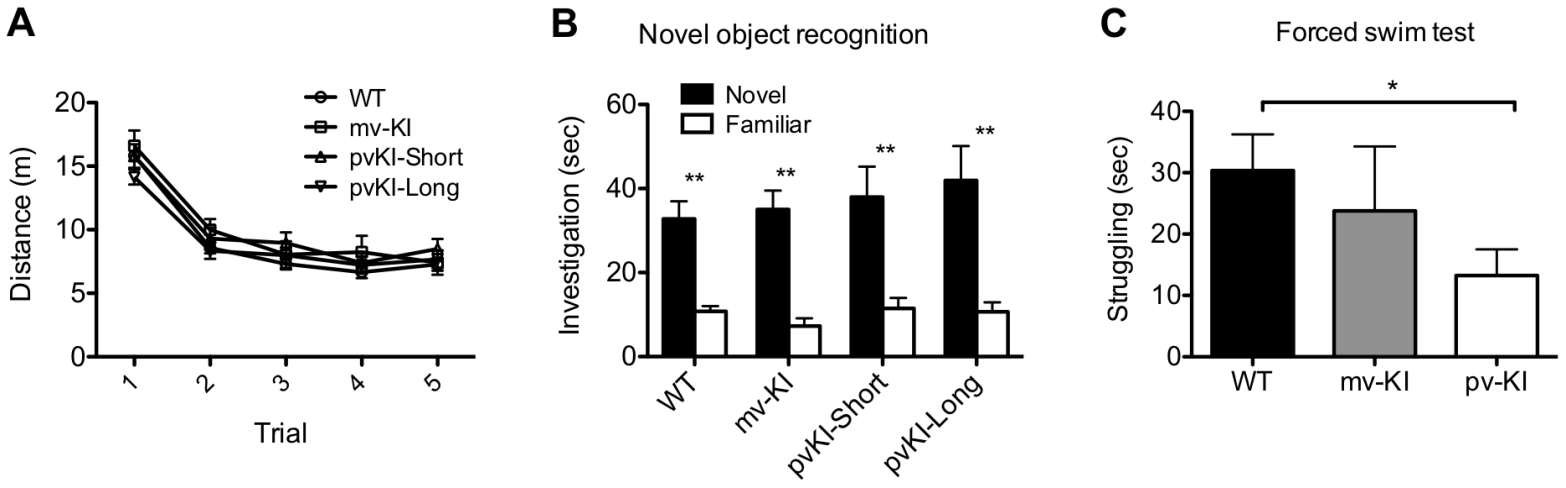
Behavioral effects of altered V1aR patterns. A) Genotype did not affect locomoter adaptation in the novel object arena (n = 38 WT, 10 mvKI, 12 pvKI-short, 20 pvKI-long). B) All groups displayed normal novel object recognition **p<0.05. C) pvKI mice, which exhibit increased levels of V1aR in the central amygdala, spend less time struggling in the forced swim test (minutes 4–6) (n = 43 WT, 31 pvKI, and 10 mvKI; *p = 0.02). Data are represented as mean ± SEM.

Novel object recognition is a hippocampus-dependent task, and performance on this task is tied to differences in excitability of the dentate gyrus [50], [51]. Thus, we hypothesized that activation of V1aR in the hippocampus, which leads to increased firing rates [52], [53], might impact novel object recognition. All groups showed normal locomoter habituation upon repeated exposure to the novel object chamber (Figure 6A). A repeated measures ANOVA with the Greenhouse-Geisser F-test revealed a main effect of trial (F(3.27, 244.5) = 196.2; p<0.001) but no interaction between trial and genotype (F(9.65, 244.5) = 1.64; p = 0.097). In addition, all groups showed a preference of the novel object during the probe trial (Figure 6B), and no group differences were observed in the percent time spent investigating the novel object (one way ANOVA; F(3, 486.4) = 1.29; p = 0.30). A repeated measures ANOVA with the Greenhouse-Geisser F-test revealed a main effect of object (F(1, 22197.6) = 55.15; p<0.001) but no interaction between object and genotype (F(3, 196.4) = 0.49; p = 0.69). Post-hoc paired T-tests with Bonferroni correction indicate that all groups preferred the novel object (WT: t(37) = 5.27, p<0.001; mvKI: t(9) = 6.77, p<0.001); pvKI-short: t(11) = 3.5, p = 0.005; pvKI-Long: t(19) = 3.67, p = 0.002).

In addition, V1aR in the CeA has been shown to modulate stress coping behavior in rats and mice. In particular, swim stress elicits release of AVP into this region, and localized V1aR receptor blockade increases the amount of time rodents spend struggling in the forced swim test [46], [47]. Thus in a separate cohort of mice, we tested stress coping behavior and hypothesized that pvKI mice (both long and short), which have higher levels of V1aR in the CeA than WT and mvKI mice, would show lower levels of active coping in the forced swim test. While struggling did not differ across WT mice from the three lines (F (42) = 0.13, p = 0.88), we found that pvKI-long and pvKI-short mice struggled less in the forced swim test than did their WT littermates (Figure 6C; t = −2.35, p = 0.02), consistent with what would be expected based on pharmacological studies.

### Mutation rates of the *Avpr1a* microsatellite

Microsatellite sequences have been hypothesized to act as evolutionary “tuning knobs,” because they mutate at faster rates than other parts of the genome, potentially due to “slippage” of the DNA polymerase while copying these highly repetitive regions. To investigate the rates of mutation in the *Avpr1a* microsatellite, we compared the sequence of the microsatellite region in 6^th^ and 7^th^ generation animals to those of the founder animals (n = 17 pvKI-long, 15 pvKI-short, and 10 mvKI microsatellite alleles from individuals born to different parents). No spontaneous mutations occurred in the intervening generations. While mutation rates are species specific, this suggests that changes in the microsatellite sequence do not occur every generation, but rather on a longer evolutionary scale, which is in accordance with previously reported mutation rates of 10^−2^ to 10^−5^ mutations per locus per generation [54].

### Differences in predicted transcription factor binding at the *Avpr1a* microsatellite

The mechanisms underlying microsatellite-mediated differences in *Avpr1a* expression are not known. Transcriptional differences may ultimately depend on a combination of differences in DNA secondary structure, epigenetic characteristics, and/or differential binding of transcriptional enhancers within the microsatellite-containing region [15]. In order to gain insight into the latter, we used the transcription factor prediction software, MatInspector, to investigate the sequences shown in Figure 1, which include both short tandem repeats and interspersed non-repetitive DNA, (Genomatix, AnnArbor, MI) [55], [56]. We used Matrix Family Library Version 8.4 to match a database containing potential binding sites of 7018 vertebrate transcription factors to our sequences. MatInspector identified 21 potential TF binding sites in the meadow microsatellite, and 160 and 141 sites in the long and short allele, respectively. These sites corresponded with 21 different transcription factors potentially capable of binding to the meadow allele, and 60 for the long allele and 59 for the short allele. Comparison of these lists indicated that 4 transcription factors putatively bind the meadow microsatellite region but not the prairie alleles. In addition, we identified 5 factors that would uniquely bind to the prairie long allele, and 2 to the short allele. In order to further focus these lists, we examined their expression profiles using the Allen Brain Atlas [57], reasoning that any transcription factor responsible for differences in *Avpr1a* expression would need to be expressed within the brain. While most transcription factors showed at least moderate levels of expression in a few brain regions, one factor that putatively binds uniquely in the long microsatellite, Ras-responsive element binding protein 1 (*Rreb1*), was particularly notable because it is highly expressed within the dentate gyrus (Figure 1a). This corresponds with the differences in V1aR levels in the DG of pvKI-long versus pvKI-short mice. It should be noted that the Ras-responsive element is located in a non-repetitive sequence and is due to a G/A single nucleotide polymorphism rather than a VNTR polymorphism.

In addition, we hypothesized that differences in the number of transcription binding sites may also be important for modulating V1aR levels. We compared the number of predicted binding sites identified in the long and short allele. Among transcription factors that putatively bind both the long and short allele, 11 factors had more potential binding sites in the short allele and 8 in the long allele. The most notable differences in the number of putative binding sites were attributable to variation in length of repetitive sequences. For instance, expansion of a GAGA tetra-nucleotide repeat in the long allele generates up to 23 additional opportunities for binding of the GAGA-binding factor, cKrox/th-POK while expansion of a TATA repeat in the short allele resulted in 6 additional binding opportunities for TATA-binding factors (Figure 1a). These analyses provide potential new avenues of research to better understand the transcription-factor based mechanisms that may underlie microsatellite-mediated differences in *Avpr1a* expression.

## Discussion

Changes in transcriptional regulation are a primary driver of phenotypic evolution [58]. Here we demonstrate that the proximal 3.4 kb of the 5′ flanking region of the rodent *Avpr1a* gene has only a modest impact on species-specific expression patterns, indicating that elements outside of this region are important for many expression differences. Further studies using targeting vectors incorporating elements downstream of that used here, including coding region, intron, and 3′ untranslated region would be useful to determine whether the species specific patterns seen in our previous transgenic mouse study were conferred by downstream elements. Studies examining the genetic regulation of oxytocin and AVP gene expression have revealed the important role of intronic or 3′ flanking regions for cell-type specific expression [59]–[63]. Alternatively, more distal 5′ flanking regions, or even chromosomal landscape may play an important role in determining species-specific expression patterns [64]. However, our data do confirm that both species differences and intra-species variation in microsatellite structure contribute to variation in gene expression. To our knowledge, this is the first demonstration that species differences and individual variation in microsatellite structure has a direct impact on the expression pattern of a behaviorally relevant gene.

Our findings support the hypothesis that the instability of genetic elements proximal to genes may act as “evolutionary tuning knobs” to enhance the evolvability of traits through alteration of gene expression [14], [65], [66]. Microsatellite sequences typically mutate at faster rates than non-repetitive DNA [67], and unlike other forms of mutations, such as SNPs and indels, expansion or contraction of a microsatellite sequence is reversible [68]. Further, addition or subtraction of repeat units can exert small, quantitative effects on gene expression levels, such as those seen in the DG of pvKI-long and pvKI-short mice, leading to high gene expression divergence in a population of individuals carrying different microsatellite alleles. Repeat variation can alter gene expression via multiple mechanisms, including differential recruitment of transcriptional enhancers, altered secondary structure (e.g. bendability) of the DNA strand, and differences in epigenetic modifications that affect nucleosome binding [15]. Our results indicate that the *Avpr1a* microsatellite affects gene expression in multiple, but not all, brain regions. Because different mechanisms may underlie microsatellite-mediated expression changes in different brain regions, the inherent flexibility of phenotype conferred by repetitive sequences may be enhanced in the complex cellular environment of the brain, as compared to a single cell organism or a more homogenous tissue. It should be noted, though, that the differences in each of our comparisons between the lines created less divergence in V1aR binding than we anticipated, suggesting that other linked polymorphisms found outside of the microsatellite, as well as outside of the 3.4 Kb 5′ flanking region are contributing to the more robust differences reported in the vole studies.

*Avpr1a* is a particularly interesting locus for understanding the genomic mechanisms of phenotypic diversity, as it has been implicated in modulating social behavior in multiple species, including humans. An initial study reported that monogamous prairie and pine voles had longer *Avpr1a* microsatellites than nonmonogamous meadow and montane voles, suggesting that the presence of the microsatellite may have contributed to the evolution of the monogamous mating strategy in voles [24]. However, a subsequent survey of the *Avpr1a* locus in several other vole species and, more recently, in *Peromyscus* species did not support the hypothesis that the presence or absence of the microsatellite element was associated with monogamy [64], [69]. Nevertheless, more subtle differences in microsatellite structure may result in inter- and intraspecies differences in receptor expression, which could contribute to species differences in the expression of behaviors associated with monogamy [17], [70].

There is conclusive evidence that variation in *Avpr1a* expression contributes to variation in social behavior [17], [32], [34]. The present findings cannot confirm that variation in the microsatellite structure contributes to variation in social behavior in mice. Indeed, it is unlikely that social behaviors are significantly affected in our knock-in mice since the greatest alteration in V1aR expression were found in regions that have not been implicated in AVP-dependent social behavior. However, our results do support the more general hypothesis that variation in the *Avpr1a* microsatellite structure directly contributes to variation in V1aR density in a brain region specific manner.

Similar VNTRs are found proximal to the primate *AVPR1A* gene, and differences in the presence and composition of these regions exists both within and between species [71]–[73]. In humans, at least 16 alleles exist for a complex microsatellite located upstream of *AVPR1A*, known as RS3 [72]. It is worth noting that the specific sequences and location of the human microsatellite are different from that found in voles, but this region represents an analogous genetic region with putatively enhanced mutation rates. Variation in the length of this region has been associated with differences in V1aR mRNA levels in post-mortem human hippocampus, similar to our findings in the prairie long and short KI lines [74]. In addition, RS3 allelic variation predicts amygdala reactivity in response to face presentation, a highly salient social stimulus for humans [75]. Genetic studies have suggested a role for variation in RS3 and other *AVPR1A* microsatellites in multiple aspects of human social behavior, including male pair bonding and relationship quality, and altruism [39], [40], [74], [76]–[78]. In addition, nominal associations between RS3 variants and autism, a disorder characterized by deficits in social behavior, have been reported [79]–[81].

Chimpanzees are polymorphic for an indel that includes RS3 [71], and the presence or absence of this VNTR-containing region is associated with differences in a variety of personality traits. In particular, males carrying the RS3-containing allele demonstrated higher levels of dominance traits and lower levels of conscientiousness than males that lacked RS3 [41]. Together, these studies suggest that microsatellite diversity affecting *Avpr1a* expression may be a general mechanism for generating behavioral diversity in primates as well as rodents.

Our results suggest that variation in the microsatellite structure of *Avpr1a* can impact expression in the brain, but only to a modest extent, at least in mice. While we did not see an effect of the microsatellite on expression in regions associated with social behavior in our mice, it is conceivable that in the context the vole or human genome, similar microsatellite variation could have a larger impact on expression in regions involved in modulating social behavior, and thus could generate variation in the expression of behavior. Our results do suggest that the regulatory elements contributing to species-specific expression patterns are not confined to the proximal 5′ flanking sequence, and the regulation of species-specific expression patterns for this gene is more complex than we originally hypothesized. Future studies replacing larger stretches of the 5′ flanking region, exons and introns, or utilizing BAC transgenics may be able to further elucidate how species-specific patterns of gene expression in the brain are achieved.

## Materials and Methods

### Ethics statement

All animal protocols were approved by the Columbia University Internal Animal Care and Use Committee and were conducted in accordance with the National Institutes of Health Guide for Care and Use of Laboratory Animals.

### Generation of *Avpr1a* KI mice

KI mice were generated using a targeting construct illustrated in Figure 2. The homology arms were amplified from a bacteria artificial chromosome (BAC) containing the C57Bl6/J *Avpr1a* locus using an enzyme mixture that includes both taq polymerase and a proof-reading polymerase (Epicentre Biotechnologies, Madison, WI). The homology arms were sequenced and the same homology arms were used in all three targeting constructs. Three versions of the prairie vole *Avpr1a* 5′ flanking region containing the meadow and prairie long and short microsatellite versions were isolated from previous expression constructs [82]. Specifically, because the three versions of the microsatellite were independently cloned into the same vector containing the prairie vole 5′ flanking region, this region for each construct was identical except for the structure of the microsatellite. This was confirmed by direct sequencing. A floxed PGK-Neo cassette was inserted upstream of the prairie 5′ flanking region and an HSV-tk cassette was placed downstream of the 3′ homology arm. The construct was linearized via digestion with *Sbf1*.

The linearized construct was sent to Ingeneious Targeting (Stonybrook, NY) where it was electroporated into hybrid C57Bl/6J/129SV embryonic stem cells. DNA from neomycin resistant/gancyclovir-sensitive clones were screened via southern blot. Specifically, genomic DNA was digested with *Acc651* and evidence of recombination was detected using a probe located upstream of the 5′ homology arm (Figure 2). This yielded a 9.5 kb band in WT and a 5.1 kb band in correctly targeted recombinant alleles. Positive recombinants were further verified using two internal southern probes, PCR, and sequencing.

Correctly targeted recombinant stem cells were injected into blastocysts by Ingenious Targeting. Offspring of the chimeras carrying the targeted allele were crossed with mice expressing EIIa-Cre recombinase on a C57Bl6/J background. Because Cre-mediated recombination in this line is not 100% efficient, offspring were screened for deletion of the PGK-Neo cassette via PCR. All three lines were then bred to C57Bl/6J background for at least 5 generations. Animals were genotyped using the following primers: 5′ TACAAGTGAGTGGGCCTTTCCTGT and 5′ GAGCCTCGCGGGAAACTCAT for the WT allele (754 bp) and 5′ AGCTCTCTTCCATGCATTCGACCA and 5′ ACAGAAGCAACAGTGACCTTCCCT for the KI allele (334 bp) (Figure 2).

### Mouse husbandry

Mice were housed in groups of 3–5 animals with mixed genotypes, had *ad libitum* access to food and water, and were maintained on a 12∶12 light∶dark cycle. Mouse lines were maintained separately and WT and KI experimental animals were derived from heterozygous breeding pairs in each line (pvKI-long^+/−^, pvKI-short^+/−^, mvKI^+/−^).

### V1aR autoradiography

N5 and N6 generation mice were euthanized between PND 60–70 via cervical dislocation followed by decapitation. Receptor autoradiography was performed as previously described [83]. Slide mounted sections at 100 mM intervals were thawed at room temperature for 1 hour, briefly fixed on 0.1% paraformaldehyde for 2 minutes, rinsed twice with 50 mM Tris buffer (pH 7.4), and incubated with 50 pM ^125^I-linear-AVP ligand [Phenylacetyl-DTyr(Me)-Phe-Gln-Asn-Arg-Pro-Arg-Tyr-NH2; Perkin Elmer, Waltham, MA] in buffer containing 50 mM Tris (pH = 7.4), 10 mM MgCl, and 0.1% BSA for 1 hour. The slides were then washed 4×5 min in 50 mM Tris buffer with 0.2% MgCl at 4°C followed by a final 30 minute rinse in the same buffer at room temperature with agitation. Slides were rinsed briefly in double distilled water and allowed to dry overnight before exposure to BioMax MR film along with an ARC146-F ^14^C standard. Multiple exposures, ranging from 18 to 72 hours, were performed to ensure all regions of interest could be evaluated within the linear range of the film. All slides were processed simultaneously.

Receptor densities were quantified by densitometry using MCID software as previously described [33]. Quantification was performed blind to genotype. Diagrammatic representative brain sections from Paxinos and Franklin (2008) were used to define anatomical regions. Briefly, for each region quantified, 3 serial sections were sampled bilaterally. Non-specific binding was calculated by selecting a background region not expressing V1aR for each section to account for potential section to section variation. Optical density was converted to pCi/region using the standard curve calculated from the co-exposed standard. Non-specific binding as subtracted from total binding to yield values for specific binding. Specific binding values were normalized to fold change relative to WT levels. Four WT animals for each line (n = 12 total) were pooled to generate a single WT group, derived from 9 independent litters from 7 breeder pairs. Eight knockin mice from each KI line were used, originating as follows: mvKI mice – 5 litters from 3 breeder pairs, pvKI-short mice – 5 litters from 4 breeder pairs, and pvKI-long mice – 6 litters from 3 breeder pairs. In each case, the groups were half male and half female. One mvKI individual was dropped from analysis of the CeA, PVThal, and DG V1aR levels due to tissue damage.

All statistical calculations are presented as mean ± SEM, and were performed in SPSS version 19. We tested for line differences by comparing WT littermates of all 3 lines (pvWT-long, pvWT-short, and mvWT; n = 4/line) using a 2-way ANOVA with line and brain region (CeA, PVthal, DG, LS, VP) as factors. We found a significant effect of brain region (F (48) = 122.2; p<0.001), but no significant effect of line (pv-long, pv-short, mv) (F (48) = 1.095; p = 0.35), and no evidence of interaction between the two (F (48) = 0.823, p = 0.56). Based on these results, WT littermates from all three lines were grouped together in subsequent analyses as the WT comparison group. To compare V1aR density in the brains of mice with different KI genotypes, we again used 2-way ANOVAs with genotype and brain region as factors. When significant main effects of genotype or interactions were observed, we conducted a simple effects analysis for genotype using a Sidak corrected α to account for multiple comparisons.

### Behavioral tests

#### Novel object recognition

N5 to N6 adult male littermates (4–5 months old) were used to assess novel object recognition. No more than 3 animals of the same genotype were used from a given litter. We used a modified version of the protocol described by Denny et al. [50]. The testing room was lit with red fluorescent light bulbs (approximately 6 lux) and testing began at least 2 hours after lights went off in the mouse room. Behavior sessions were recorded with a video camera affixed to the ceiling. The testing arena was a standard rat cage (25.9 wide×47.6 long×20.9 cm high) with pine shave bedding with white paper affixed to the sides so that mice could not contact or see one another during testing. Mice were transported into the room in their home cages. They were singly housed 30 minutes before the test and in between trials.

Novel objects consisted of (1) a blue, ceramic shoe (diameter 9.5 cm, maximal height 6 cm); a black plastic box (8×3×9.5 cm); and a clear plastic funnel (diameter 8.5 cm). The mouse could not displace these objects, and the objects were tested previously and elicited the same levels of exploration [50]. The objects and their placements were fully randomized.

Novel object consisted of five 5 minute exposures with 3 minute inter-exposure intervals. Mice were place in the center of the arena at the start of each exposure. In between tests, mice were returned to holding cages while the arena was cleaned with a 1% Sparkleen solution and the bedding was replaced. Exposures 1–4 were habituation sessions in which two objects place symmetrically on either side of the arena ∼5 cm away from the wall. In exposure 5, one of the objects was replaced with a novel object. This is referred to as the probe trial. All mice were returned to their home cages following the 5^th^ session. All animals in the same cage were tested at the same time, and the cage order randomized with respect to the three lines. Testing for all animals was completed within an 8 day period.

Total locomotion was calculated via automated tracking using AnyMaze Software (Stoelting). Object investigation during the probe trial was scored by an observer blind to genotype using Noldus Observer. Object investigation was defined as orientation of the head toward the object with the nose within 1 cm of the object. Investigation was not scored if the mouse was on top of the object or completely immobile. Novelty preference was determined using a two way ANOVA of Object×Genotype with Object as a repeated measure.

#### Forced swim test

N6 to N7 adult male WT and KI littermates (5–6 months old) were used in the Forced Swim test. No more than 3 animals of the same genotype were used from a given litter. Since WT animals between the different lines did not differ in V1aR binding in the CeA or in time struggling (1- Way ANOVA, F (42) = 0.13, p = 0.88), all WT animals (n = 17 short WT, 14 meadow WT, 12 long WT) were combined into one group. Additionally, since pvKI-long (n = 18) and pvKI-short (n = 13) did not differ in the amount of time spent struggling (t = −0.08, p = 0.936) or in V1aR binding in the CeA, these groups were combined into a single pvKI group. Testing was performed beginning 1 hr after lights on. All animals in the same cage were tested at the same time in three to five separate swim chambers, and the cage order was randomized with respect to the three lines. Testing for all animals was completed in a single day. Behavioral response to forced swimming was measured as described in David et al. [17]. Mice were placed in clear glass buckets 20 cm deep, filled 2/3 of the way with 24–26°C water and videotaped from the side. The last four minutes of the test were scored by an observer blind to genotype using Noldus Observer. Struggling was defined as the animal moving all four limbs to swim or to attempt to crawl up the side of the container. A Student's t-test was used to test the a priori hypothesis that pvKI mice struggled more than their WT littermates.

#### Amplification of *Avpr1a* microsatellite

Genomic DNA was purified from tail tissue samples using the DNeasy purification kit (Qiagen, Valencia, CA). The V1a microsatellite region was amplified as previously described. PCR products were gel purified and extracted using the Zymoclean gel DNA recovery kit (Zymo Research, Irvine, CA). Sequencing of PCR products was performed by Macrogen USA (New York, NY).
